# A Chest Wall Myofibroma in an Adult

**DOI:** 10.70352/scrj.cr.24-0156

**Published:** 2025-04-02

**Authors:** Yasuaki Kubouchi, Tomohiro Haruki, Toho Wada, Masaya Yamasaki, Kengo Yasuda, Wakako Fujiwara, Karen Makishima, Tatsuya Miyamoto, Shinji Matsui, Yoshihisa Umekita, Masanori Hisaoka, Yugo Tanaka

**Affiliations:** 1 Department of Surgery, Division of General Thoracic Surgery and Breast and Endocrine Surgery, Faculty of Medicine, Tottori University, Yonago, Tottori, Japan; 2 Department of Pathology, Division of Pathology, Faculty of Medicine, Tottori University, Yonago, Tottori, Japan; 3 Department of Pathology and Oncology, University of Occupational and Environmental Health, Kitakyushu, Fukuoka, Japan

**Keywords:** myofibroma, myofibromatosis, chest wall tumor, adult patient

## Abstract

**INTRODUCTION:**

Myofibroma is a rare benign tumor that often involves the skin, subcutaneous tissue, and/or bone in the head and neck, typically developing in children. Adult cases, particularly in the chest wall, are extremely rare.

**CASE PRESENTATION:**

A 22-year-old asymptomatic Chinese man presented with an abnormal chest X-ray showing a shadow in the left lung apex. Chest computed tomography (CT) revealed a 2.5 × 2.0 cm nodule infiltrating the second rib. Positron emission tomography showed an accumulation of ^18^F-fluorodexyglucose in the nodule. A CT-guided biopsy revealed spindle-shaped cells positive for alpha-smooth muscle actin (α-SMA), β-catenin, and p16 but was inconclusive for malignancy. Since a malignant tumor could not be ruled out, the decision was made to perform surgery. A 25 cm-high posterolateral incision was made, the latissimus dorsi muscle was preserved, and the trapezius and rhomboid muscles were split in the direction of their fibers to reach the bony thorax. The second rib and the chest wall tumor, including the first and second intercostal muscles, were resected with a 1.5 cm margin. The lesion involving the rib showed fibromyxoid/fibrosclerotic nodules with spindle cells in loose fascicles or whorls, hypercellular areas, and thin-walled hemangiopericytoma-like vessels. Immunohistochemistry was positive for α-SMA, NOTCH3, and PDGFR and negative for desmin and S100. Based on these findings, we diagnosed a myofibroma in the chest wall.

**CONCLUSIONS:**

This case demonstrates that myofibroma can occur in the chest wall of an adult. The imaging findings of myofibromas are similar to those of malignant tumors, making them difficult to diagnose. Resection offers a good prognosis, and because these tumors occur in young patients, surgery should be performed with consideration for preserving function.

## Abbreviations

α-SMA
: alpha-smooth muscle actin
CT
: computed tomography
FDG
: fluorodeoxyglucose
PET
: positron emission tomography
SUV
: standardized uptake value

## INTRODUCTION

Myofibroma is a rare benign tumor that arises in the skin, subcutaneous tissue, and/or bone of the head and neck, typically observed in children <2 years old.^1,2)^ Myofibroma occurring on the chest wall in an adult is very rare. We describe such a tumor and its successful treatment.

## CASE PRESENTATION

**Fig. 1:**
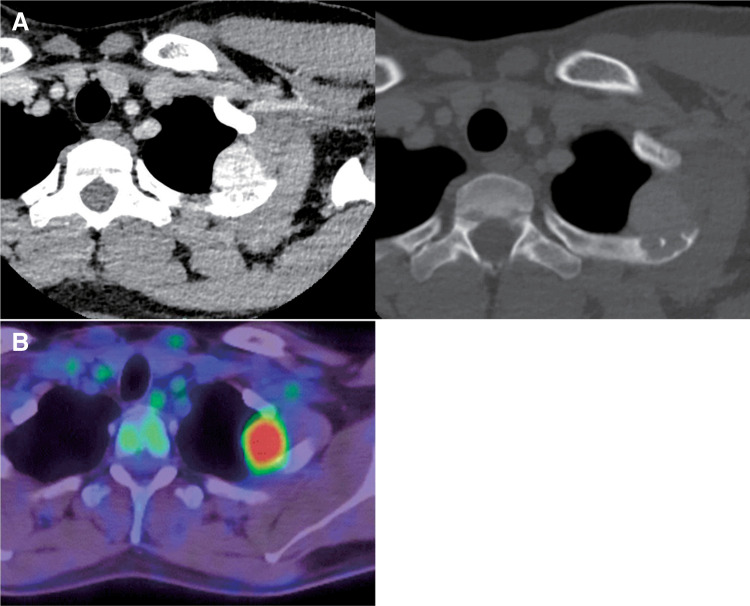
(**A**) Chest CT visualized a 2.5 × 2.0 cm nodule with a clear border spreading to the left first intercostal muscle and second rib. The nodule showed contrast enhancement. (**B**) PET revealed FDG accumulation in the nodule, with a maximum SUV of 8.48. CT, computed tomography; PET, positron emission tomography; FDG, fluorodeoxyglucose; SUV, standardized uptake value

During a medical examination, chest radiography revealed an abnormal shadow was observed in the left apical region of the lung of a 22-year-old Chinese man with no history of tobacco smoking. He had no specific medical history and was asymptomatic. A chest computed tomography (CT) scan demonstrated a 2.5 × 2.0 cm nodule with a clear border spreading to the left first intercostal muscle and second rib; the nodule showed contrast enhancement (**Fig. 1A**). Positron emission tomography (PET) revealed an accumulation of ^18^F-fluorodexyglucose (FDG) in the nodule, with a maximum standardized uptake value (SUV) of 8.48 (**Fig. 1B**).

A CT-guided biopsy was performed on the nodule. The subsequent microscopic examination revealed a proliferation of small spindle-shaped cells in a fibromucous stroma. Tumor cells were positive for alpha-smooth muscle actin (α-SMA), β-catenin, and p16, and negative for desmin, S100, STAT6, and MUC4, with a Ki-67 index of 5%. Deep fibrous histiocytoma and solitary fibrous tumor were considered in the differential diagnoses, but it was difficult to determine whether the tumor was benign or malignant based on the biopsy alone.

**Fig. 2:**
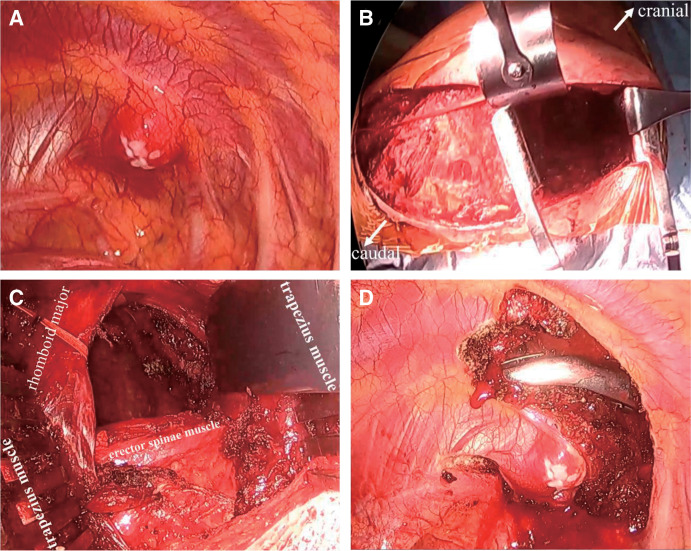
(**A**) The chest wall tumor was covered by the pleura, with no invasion into the lungs. (**B** and **C**) A 25 cm-high posterolateral incision was made, the latissimus dorsi muscle was preserved, and the trapezius and rhomboid muscles were split in direction of their fibers to reach the bony thorax. (**D**) The chest wall tumor extending from the second rib to the first and second intercostal muscles was resected with a 1.5 cm margin.

Since a malignant tumor could not be ruled out, the decision was made to perform surgery. A 5 mm thoracoscope was inserted between the 6th intercostal space, and the thoracic cavity was observed. The chest wall tumor was covered by the pleura with no invasion into the lungs (**Fig. 2A**). Grossly, we strongly suspected a benign tumor and considered that a wide excision was unnecessary; thus, a 25 cm-high posterolateral incision was made, the latissimus dorsi muscle was preserved, and the trapezius and rhomboid muscles were split in the direction of their fibers to reach the bony thorax (**Figs. 2B** and **2C**). The chest wall tumor extending from the second rib to the first and second intercostal muscles was resected with a 1.5 cm margin (**Fig. 2D**). Because of the small extent of the chest wall resection, chest wall reconstruction was not performed. The operative time was 122 min, and the blood loss was 60 g. The patient was discharged on the 6th postoperative day without complications.

**Fig. 3:**
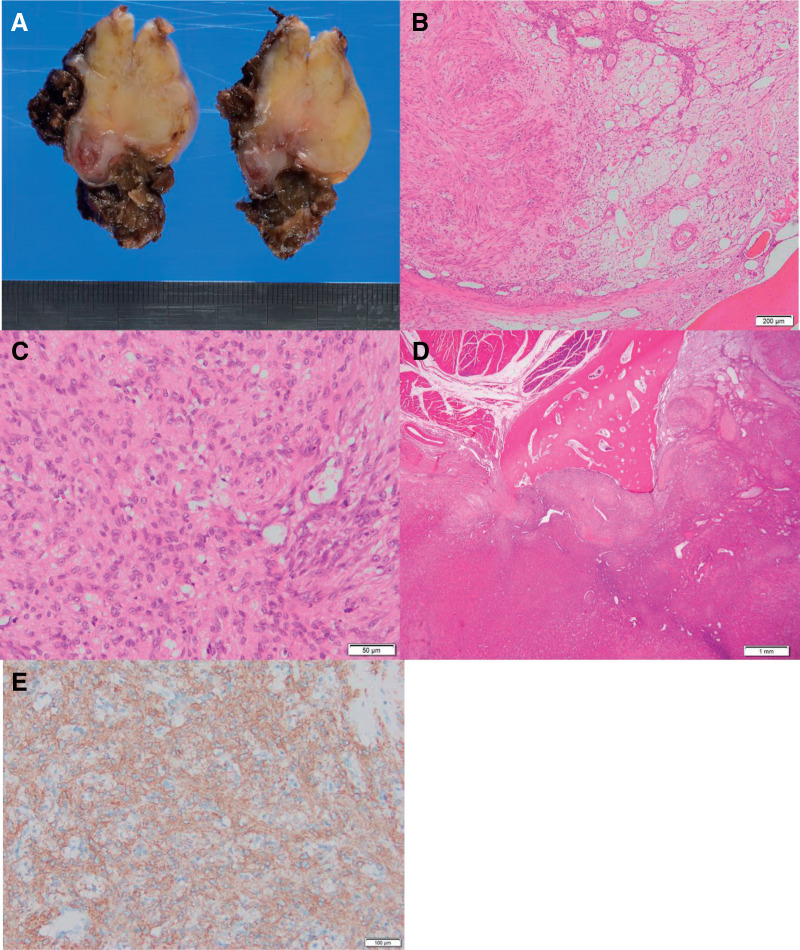
(**A**) The excised specimen was a relatively well-defined lobulated mass measuring 32 × 27 × 24 mm in size. (**B** and **C**) Histopathological examination revealed fibromyxoid or fibrosclerotic nodules of proliferating myoid spindle cells arranged in loose fascicles, whorls, or haphazardly, alternating with hypercellular areas of oval or short spindle cells with viably gaping thin-walled blood vessels, including a hemangiopericytomatous form. (**D**) The tumor had compressively osteolyzed the rib cortex and extended into the bone marrow. (**E**) Immunohistological examination demonstrated α-SMA positivity. α-SMA, alpha-smooth muscle actin

The excised specimen was a relatively well-defined lobulated mass measuring 32 × 27 × 24 mm in size (**Fig. 3A**). Histopathological examination showed that the nearly well-circumscribed multinodular or lobular lesion, focally involving the rib bone, was composed of fibromyxoid or fibrosclerotic nodules. There was no mitosis or necrosis. Proliferating myoid spindle cells were arranged in loose fascicles, whorls, or haphazardly, alternating with hypercellular areas of oval or short spindle cells with thin-walled blood vessels, including a hemangiopericytomatous form (**Figs. 3B** and **3C**). The tumor had compressively osteolyzed the rib cortex and extended into the bone marrow. The margins of the osteolyzed rib cortex were smooth, with no destructive extension, and there was no inflammatory cell infiltration in the surrounding area (**Fig. 3D**).

The immunohistological examination revealed positivity for α-SMA (**Fig. 3E**) and showed that the tumor cells were diffusely positive for NOTCH3 and PDGFR in the cytoplasm. Desmin, S100, and CD34 were negative. Based on these results, the tumor was diagnosed as a myofibroma.

The patient was followed up without additional treatment and has survived for >1 year without recurrence. He provided his fully informed written consent for the publication of this report and case images.

## DISCUSSION

Myofibroma and myofibromatosis are benign mesenchymal neoplasms composed of myofibroblasts and are classified as perivascular tumors in the 2020 World Health Organization (WHO) classification of bone and soft tissue tumors.^3)^ The term “myofibroma” is used to refer to solitary lesions, and “myofibromatosis” is used to refer to multicentric lesions. Approximately 90% of myofibromas occur in children, and 65% of the cases are observed during the first 2 years of life,^1)^ mostly in the soft tissues of the head, neck, and trunk.^4)^ The exact etiology of myofibroma is unknown, but the possibility of familial inheritance, either autosomal dominant or recessive, has been suggested.^1)^ Myofibromas usually occur in the skin, superficial soft tissues (head, neck, and trunk), and bone,^3)^ but have also been reported in visceral organs such as the liver and pancreas.^5)^ Myofibromas that occur in deep soft tissues of the chest wall, as in the present patient, are extremely rare. Our search of the relevant literature indicates that our patient's case provides only the second report of a myofibroma in the chest wall.

Chen et al. described a painful myofibroma in the chest wall in a patient in his 20s in 2014.^6)^ Our patient exhibited osteolytic changes in the ribs, but these changes were not accompanied by pain. The absence of pain could be attributed to the fact that the osteolytic changes were caused by long-term compression of the tumor and were not accompanied by symptoms, unlike bone invasion by a malignant tumor. Myofibroma in the jaw generally shows destruction of cortical bone and has been reported to mimic odontogenic tumors and malignant neoplasms,^7)^ and the myofibroma of rib origin in the present patient showed similar findings.

There are few reports of PET findings regarding myofibroma, but Shibuya et al. described a hyperaccumulation of FDG in myofibroma of the mandible.^8)^ A PET examination revealed FDG hyperaccumulation in our patient as well, even though the tumor was benign; the mechanism of this hyperaccumulation is unknown. Extra caution should be exercised when osteolytic changes and hyperaccumulation of FDG are observed, as these cases must be differentiated from malignancy.

Histologically, a myofibroma is a well-defined nodule with a bilaminar pattern. The center is characterized by round- to spindle-shaped tumor cells and hemangiopericytoma-like branching vessels. The margins are characterized by nodular to bundle-like structures of vitellogenic cells, muscle-like cells, and cartilage-like cells. Immunohistological examination is often positive for α-SMA, vimentin, and HHF-35, while CD34, S100, and desmin are often negative.^7)^ The search for causative genes has progressed, and the PDGFRB gene and NOTCH3 have been reported as causative genetic mutations, which can contribute to the diagnosis.^9)^

Surgical treatment of myofibroma is common and has good results. We performed surgical resection in the present case, but because the tumor was benign and the patient was young, we tried to avoid muscle dissection as much as possible. A high posterolateral skin incision was made, and the trapezius and rhomboid muscles were split in the direction of their fibers to reach the bony thorax without muscle dissection. Although the visual field was somewhat poor because of the lack of muscle dissection, the resection was possible because of the tumor's small size. If a similar tumor is malignant, the trapezius and rhomboid muscles should be transected for adequate margins, and the resection should be performed with a good visual field. However, function-preserving surgery should be performed based on the patient's background and oncological prognosis.

## CONCLUSIONS

We have described an extremely rare case of a myofibroma occurring in the chest wall of an adult. The imaging findings of myofibromas are similar to those of malignant tumors, making these tumors difficult to diagnose. Resection offers a good prognosis, and because these tumors occur in young patients, surgery should be performed with consideration for preserving function.

## DECLARATIONS

### Funding

No funding was received for this study.

### Authors’ contributions

YK administered the clinical treatments and drafted the manuscript.

YT conducted a detailed review and contributed to the discussion.

WF, TM, SM, KM, MH, and YU assisted in making the pathological diagnosis.

All authors read and approved the final manuscript and take full responsibility for its content.

### Availability of data and materials

The datasets supporting the conclusions of this article are included within the article.

### Ethics approval and consent to participate

This work does not require ethical considerations or approval.

### Consent for publication

Fully informed written consent was obtained from the patient for the publication of his case and images. His identity has been protected.

### Competing interests

The authors declare that they have no competing interests.
